# Stability of active zone components at the photoreceptor ribbon complex

**Published:** 2010-12-12

**Authors:** Hanna Regus-Leidig, Dana Specht, Susanne tom Dieck, Johann Helmut Brandstätter

**Affiliations:** 1 Department of Biology, Animal Physiology, University of Erlangen-Nuremberg, Erlangen, Germany; 2 Max Planck Institute for Brain Research, Department of Synaptic Plasticity, Frankfurt/Main, Germany

## Abstract

**Purpose:**

Photoreceptor ribbon synapses translate light-dependent changes of membrane potential into graded transmitter release over several orders of magnitude in intensity. A specialized organelle at the active zone – the synaptic ribbon – is a key player in this process, and it is well known that the ribbon undergoes illumination and thus activity-dependent structural changes. However, the molecular basis for these changes is unknown. The aim of this study was to correlate the known ultrastructural ribbon changes to the distribution of proteins of the presynaptic ribbon complex.

**Methods:**

In an in vitro assay, two distinct structural ribbon states – club-shaped and spherical-shaped – were enriched and the distribution of presynaptic proteins at the rod photoreceptor ribbon complex was analyzed with immunocytochemistry and light and electron microscopy.

**Results:**

We show that structural changes of the ribbon correlate with the redistribution of selected presynaptic proteins. The disassembly of the ribbon complex seems to be a multistep process, which starts with the removal of spherical ribbon material while arciform density and active zone plasma membrane proteins remain largely unchanged at their synaptic location. Only later, in a second phase following the removal of ribbon material, the arciform density and plasma membrane proteins are redistributed from their synaptic localization and active zones disappear.

**Conclusions:**

The results of our study show that photoreceptor ribbon and arciform density/plasma membrane components might be influenced differentially by activity-driven processes, thus providing a molecular basis for further investigation of regulatory and adaptive processes in photoreceptor ribbon synaptic transmission.

## Introduction

The retina receives light signals over several orders of magnitude. Adaptive changes to different light levels occur at multiple sites within retinal signal transmission and act together in processing the light information. A change in synaptic strength is one putative mechanism involved [1–6].

In contrast to conventional neurons, photoreceptors do not signal via action potentials but continuously translate light into graded transmitter release, with highest exocytosis rates in the dark [7]. To accomplish this task, photoreceptors as well as retinal bipolar cells contain a specialized type of synapse, the so-called ribbon synapse. The synaptic ribbon, a prominent cytomatrix structure at the photoreceptor active zone covered by hundreds of synaptic vesicles, is thought to play a role in regulating synaptic transmission, although its exact contribution is still a matter of debate [7–14].

In the mammalian retina, ribbons are plate-like structures with a width of 30 to 50 nm. Their number and shape depends on the cell type: cone photoreceptor and bipolar cell terminals possess several release sites with small ribbon plates while rod photoreceptor terminals usually contain a single large ribbon that bends around the invaginating postsynaptic elements at a length of 1–2 µm, forming a horseshoe-like structure which is already visible at the light microscopic level [15].

Photoreceptor ribbons are highly dynamic structures, and daytime (circadian), illumination dependent or pharmacologically triggered changes in ribbon size, shape and number have been described in various ribbon-containing cell types, such as photoreceptor and bipolar cells, and species such as mouse, rat, and fish [1,3,5,6,16–19]. In an interesting study, Balkema and colleagues [3] examined the relationship between variations in ribbon length and visual function in normal and albino mice during the diurnal cycle. Dark-adapted visual thresholds were determined behaviorally with a water-maze assay. For normal and albino mice, the lowest visual threshold, i.e., the highest sensitivity, corresponded to the time when ribbons were the longest. The highest visual threshold, i.e., the lowest sensitivity, corresponded to the time when ribbons were the shortest. Furthermore, the visual thresholds of albino mice were elevated compared to normal mice at all time points tested, a result corroborated by the fact that ribbons in normal mice were on average longer than those in albino mice. This study clearly demonstrated that ribbon length and visual sensitivity vary together in a diurnal cycle [3].

A potential mechanism for ribbon structural changes was described in ultrastructural studies by Adly et al. [1] and by Spiwoks-Becker et al. [5]. In response to changing illumination – increase or decrease in light intensity – rod photoreceptor ribbons lose or regain material, respectively. With increasing illumination, spherical ribbon material pinches off the rod photoreceptor ribbons, leading to club-shaped profiles and free-floating spheres [5]. Local reuse of the spherical ribbon material was suggested to underlie the subsequent elongation of ribbons in darkness [1,5]. Recently, it was also shown that the protein composition of ribbons in the pineal organ is regulated by illumination [20].

Although it became clear from the mentioned studies that the ribbon is a dynamic organelle, the molecular basis of these changes is still unknown. In addition, it remains unknown whether the changes described to date can be explained by a common mechanism. To better understand the nature of the proteins taking part in the structural changes at the photoreceptor ribbon synapses, we experimentally induced the disassembly of the presynaptic ribbon complex, and analyzed the distribution of presynaptic proteins at the rod photoreceptor ribbon complex using immunocytochemistry and light and electron microscopy. We found that ribbon complex disassembly occurs in at least two phases. The first phase is characterized by the removal of spherical ribbon material, while arciform density and active zone plasma membrane proteins remain stably localized at their synaptic location. Only later, in a second phase following the removal of ribbon material, the arciform density and plasma membrane proteins are redistributed from their synaptic localization and active zones disappear.

## Methods

### Animals

All animal experiments were performed in compliance with the guidelines issued by the University of Erlangen-Nuremberg and the Max Planck Society. Thirty C57BL/6 mice at the age of 7 to 9 weeks were used in this study. The mice were kept under a 12/12 light/dark cycle with light onset at 06:00 AM; average illumination 50 lux.

### In vitro assay to enrich spherical ribbon material in C57BL/6 mice

Mice were anesthetized with isofluorane and decapitated to remove the eyes. The retinae were rapidly removed from the eyes using the Winkler method [21] and incubated for 30 min at 27 °C in 300 µl oxygenated Hanks medium (CaCl_2_ 1.3 mM, MgCl_2_ 1.5 mM, KCl 3.1 mM, HEPES 10 mM, NaCl 137 mM, D-Glucose 10 mM) with either 1 µM Ca^2+^ ionophore A23187 (Calbiochem, Darmstadt, Germany) or 10 mM EGTA. Further experiments were performed under the same conditions with membrane permeable analogs, 1 mM EGTA-AM or 1 mM BAPTA-AM, and led to similar effects (not shown). Retinae were subsequently fixed for 30 min in 4% (w/v) paraformaldehyde in phosphate buffer (PB; 0.1 M, pH 7.4). The retinae were cryoprotected in increasing concentrations of sucrose in PB (10%, 20%, 30%) at 4 °C before being frozen in a freezing medium (Reichert-Jung, Bensheim, Germany) as sandwiches of treated and untreated retinae, which were immediately fixed after removal. Vertical sections of 16 µm thickness were cut on a cryostat (Leica Microsystems, Nussloch, Germany), collected on slides and stored at –20 °C.

Immunocytochemical labeling was performed using the indirect fluorescence method. The retinal sections were blocked for 1 h in blocking solution (10% normal goat serum, 0.5% Triton X-100 in PB) and incubated in the primary antibodies (diluted as indicated in Table 1 in 3% normal goat serum, 0.5% Triton X-100 in PB) overnight at room temperature. After being washed three times in PB, sections were incubated with secondary antibodies coupled to AlexaFluor 488 (green fluorescence) or AlexaFluor 594 (red fluorescence) for 1 h at room temperature in the dark (1:500; Molecular Probes; Eugene, OR). Images were taken with a Zeiss confocal laserscanning microscope (LSM5 Pascal; Zeiss, Oberkochen, Germany) and a Zeiss Axio Imager.Z1 equipped with an ApoTome (Zeiss, Oberkochen).

**Table 1:** Primary antibodies used in light and electron microscopy

**Antibody**	**Antigen specificity**	**Dilution/ species**	**Source and catalog number**	**Reference**
Bassoon	Amino acids 756–1,001 of rat Bassoon	1:2,500 Mouse, monoclonal	Stressgen, Victoria, Canada; VAM-PS003E Clone SAP7F407	[27,29,35]
Bassoon sap7f	Amino acids 756–1,001 of rat Bassoon	1:16,000 Rabbit, polyclonal	E. D. Gundelfinger, Leibniz Institute for Neurobiology, Magdeburg, Germany	[27,31,35]
Cacna1f (Pep3)	Peptide sequence AEEGRAGHRPQLSELTN, located in the cytoplasmic loop between domains I and II of mouse Cacna1f	1:5,000 Rabbit, polyclonal	Marion Maw, Otago University, New Zealand	[29]
CtBP1	Amino Acids 345–441 of mouse CtBP1	1:5,000 Mouse, monoclonal	BD Biosciences, Heidelberg, Germany; 612042	[27]
				
Kinesin II KIF3A	Against the 130 kDa heavy chain of sea urchin Kinesin	1:100 Mouse, monoclonal	COVANCE/BabCO, Richmond, CA, USA; MMS-1 88P	[27]
				
Piccolo 44a	Amino Acids 2,172–2,361 of rat Piccolo	1:2,000 Guinea pig, polyclonal	E. Gundelfinger, Leibniz Institute for Neurobiology, Magdeburg, Germany	[23,27,29–31,35]
RIBEYE/ CtBP2	Amino Acids 361–445 of mouse RIBEYE B domain/CtBP2	1:10,000 Mouse, monoclonal	BD Biosciences, Heidelberg, Germany; 612045	[23,27,29,31,35]
RIBEYE	aa 101–207 of rat Ribeye A domain	1:1,000 Rabbit, polyclonal	Synaptic Systems, Göttingen, Germany; 192103	[30]
				
RIM 1	Amino acids 596–705 of rat RIM1 containing part of PDZ domain	1:1,000 Rabbit, polyclonal	Synaptic Systems, Göttingen, Germany; 140003	this study
RIM 2	Amino acids 925 – 1,114 of rat RIM 2	1:1,000 Rabbit, polyclonal	Synaptic Systems, Göttingen, Germany; 140303	this study
Veli3	Synthetic peptide derived from the C-terminus of the rat Veli3 (MALS-3) protein	1:1,000 Rabbit, polyclonal	Zymed, San Francisco, CA, USA; 51–5600	[28,29]

### Retinal tissue preparation for conventional electron microscopy and electron microscopic immunocytochemistry

For pre-embedding immunoelectron microscopy, the retinae were processed using a few adaptations according to established procedures [22,23]. Briefly, the retinal tissue was dissected and incubated as described for the in vitro assay, then cryoprotected and submitted to three freeze/thaw cycles. Retina pieces were embedded in agar, and 50 µm thick vertical sections were cut with a vibratome (VT 1000S; Leica, Wetzlar, Germany). The pre-incubation step was performed in blocking medium without Triton X-100. Tissue sections were incubated in primary antibody dilutions (Table 1) for 4 days at 4 °C. Biotinylated goat anti-rabbit or goat anti-mouse IgGs were applied as secondary antibodies for 2 h (1:100; Vector, Burlingame, CA). To visualize the antibody binding, a peroxidase-based enzymatic detection Kit (Vectastain Elite ABC kit; Vector) was used according to the manufacturer’s instructions. The post-fixation was performed with 2.5% (v/v) glutaraldehyde in cacodylate buffer for 1 h. The reaction product was silver intensified. Prior to the dehydration using an ethanol series and propylene oxide, the sections were incubated in 0.5% osmium tetroxide for 30 min at 4 °C. Sections were embedded in Epon resin (Fluka, Buchs, Switzerland). The ultrathin sections were stained with uranyl acetate and lead citrate.

For conventional electron microscopy, the retinae were immersion fixed with 2.5% glutaraldehyde and 4% paraformaldehyde for 2 h. The contrasting was performed in potassium ferrocyanide and 2% osmium tetroxide in cacodylate buffer. Specimens were dehydrated using an ethanol series and propylene oxide with 0.5% (w/v) uranyl acetate added at the 70% ethanol step. Final steps were performed as described for pre-embedding immunoelectron microscopy.

The ultrathin sections were examined and photographed using a Zeiss EM10 electron microscope (Zeiss, Oberkochen) and a Gatan SC1000 OriusTM CCD camera (GATAN, Munich, Germany) in combination with the DigitalMicrographTM software (GATAN, Pleasanton, CA).

### Quantification

**Figure 1:**
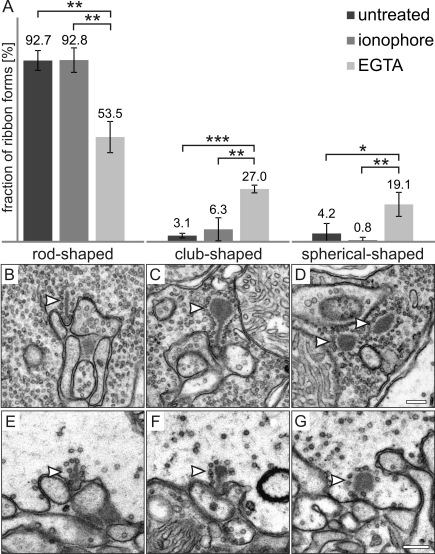
Enrichment of club-shaped and spherical-shaped ribbon material using EGTA treatment. **A**: The percentage of rod-shaped ribbons is significantly reduced in EGTA-treated retinas (EGTA) compared to ionophore A23187-treated or untreated retinas, while the percentage of club-shaped and spherical-shaped ribbon profiles is significantly increased (n=237 ribbon profiles for EGTA- and ionophore-treated retinas; n=294 ribbon profiles for untreated retinas; error bars=SD). **B**-**G**: Electron micrographs of rod (**B**, **C**, **D**) and cone (**E**, **F**, **G**) photoreceptor terminals, showing examples of different ribbon states (arrowheads): rod-shaped (**B**, **E**), club-shaped (**C**, **F**), and spherical-shaped (**D**, **G**). * p<0.05, ** p<0.01, *** p<0.001. Scale bars: 0.2 µm.

For the quantification of synaptic ribbon states (Figure 1), ultrathin sections of retinae prepared for conventional electron microscopy were evaluated (n=3 animals per condition). Series of at least 50 images were taken from the outer plexiform layer (OPL) so that a minimum of 200 rod photoreceptor terminals were evaluated per condition. The rod photoreceptor terminals, which contained ribbon material, were grouped into one of the following three categories: (1) terminals with rod-shaped ribbon material, (2) terminals with club-shaped ribbon material, and (3) terminals with spherical-shaped ribbon material. Statistical analysis was performed using the unpaired *t*-test in SigmaStat 2.0 (SPSS Inc., Chicago, IL).

Quantification of Bassoon/RIBEYE co-localization was performed on micrographs of vertical sections of EGTA-treated and untreated retinae (n=6 animals per condition). Using confocal light microscopy, three image stacks of retinae from every animal were taken and projected. Horseshoe-shaped Bassoon or RIBEYE stained profiles in rod photoreceptor terminals were counted for every animal, taken as 100% and checked for the morphological appearance of the respective counterpart staining. Horseshoe-shaped Bassoon staining was evaluated for RIBEYE appearance, and horseshoe-shaped RIBEYE staining for Bassoon appearance.

## Results

Many transgenic mouse lines, including those with a potential value in examining molecular mechanisms underlying structural and functional ribbon changes, are on a C57BL/6 background. In this study, we artificially enriched spherical ribbon material in C57BL/6 retina to correlate the changes in ribbon structure with the redistribution of proteins at the presynaptic ribbon complex.

### Enrichment of spherical ribbon material

For the first step of analyzing the molecular composition of spherical ribbon material, we searched for conditions to enrich synaptic spheres in photoreceptor terminals of C57BL/6 retina. We adapted an in vitro assay described by Spiwoks-Becker and colleagues [5] to our needs and examined the retinae using conventional electron microscopy. The retinae were rapidly dissected and either fixed directly or incubated for 30 min under high Ca^2+^ (ionophore A23187) or low Ca^2+^ (EGTA) conditions before fixation and processing for conventional electron microscopy. In randomly chosen ultrathin sections (3 animals per condition, 50 frames per animal), the rod photoreceptor terminals containing ribbon material (n>200 terminals/condition) were counted and grouped into one of the following categories: terminals with rod-shaped ribbon material (Figure 1A,B), terminals with club-shaped ribbon material (Figure 1A,C), and terminals with spherical-shaped ribbon material (Figure 1A,D).

EGTA treatment caused a significant reduction in rod-shaped ribbon material when compared to untreated and ionophore-treated retinae: untreated 92.7%, ionophore-treated 92.8%, and EGTA-treated 53.5% (Figure 1A). In accordance with this finding, the frequency of club-shaped and spherical-shaped ribbon material was significantly increased in the EGTA-treated retinae: untreated 3.1%, ionophore-treated 6.3%, and EGTA-treated 27.0% (club-shaped ribbon material); untreated 4.2%, ionophore-treated 0.8%, EGTA-treated 19.1% (spherical-shaped ribbon material; Figure 1A). In addition, the number of rod photoreceptor terminals with postsynaptic invaginations but no synaptic ribbons was significantly increased in EGTA-treated retinae versus untreated retinae (17.5% versus 6.1%; p=0.002).

Similar structural ribbon changes under low Ca^2+^ conditions, i.e., an increase in the frequency of club-shaped and spherical-shaped ribbon material, could also be observed in cone photoreceptor terminals of EGTA-treated retinae (Figure 1E-G). It must be pointed out, however, that cone photoreceptor terminals were much more sensitive to the EGTA treatment than rod photoreceptor terminals. This frequently resulted in pathologically enlarged, electron-lucent terminals. Therefore, cone photoreceptor terminals and their synapses were excluded from further analysis.

In conclusion, the EGTA treatment of the retina (low Ca^2+^) led to significant structural changes in ribbon states in the photoreceptor terminals. We were then able to use these experimentally triggered structural changes to further analyze the identity of the presynaptic proteins participating in these changes.

### Light microscopical analysis of changes in the ribbon state

**Figure 2:**
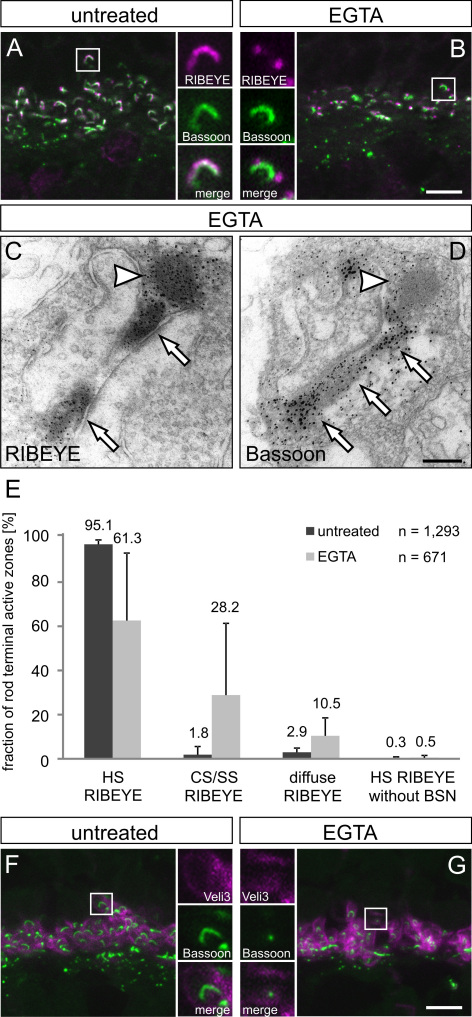
Bassoon localization at the rod photoreceptor ribbon complex is more stable than RIBEYE localization. **A, B**: Representative images of the outer plexiform layer (OPL) with the photoreceptor ribbon synapses double labeled for RIBEYE (magenta) and Bassoon (green) from untreated (**A**) and EGTA-treated (**B**) retinae. Horseshoe-shaped RIBEYE lines the horseshoe-shaped Bassoon in untreated retina (**A**). In EGTA-treated retina, RIBEYE often disintegrates into several aggregates (**B**). **C**, **D**: Immunoelectron micrographs of rod photoreceptor ribbon synapses from EGTA-treated retina. RIBEYE labeling (**C**) is found at the spherical material pinching off the ribbon (arrowhead) as well as at the active zone (arrows). In contrast, Bassoon labeling (**D**) is concentrated at the ribbon base (arrows) and the material pinching off the ribbon is not stained for Bassoon (arrowhead). **E**: Quantification of the Bassoon/RIBEYE co-localization in untreated and EGTA-treated retinae. In double stained vertical sections, either horseshoe-shaped (HS) RIBEYE or horseshoe-shaped Bassoon profiles were counted and checked for the morphological appearance of the respective other marker. When counts were based on horseshoe-shaped Bassoon (bar groups 1 to 3), the fraction of sites where RIBEYE staining appears in a horseshoe shape decreases under EGTA conditions, whereas the punctate (club-shaped, CS, or spherical-shaped, SS) and cloudy appearance of RIBEYE staining increases concomitantly. When counts were based on horseshoe-shaped RIBEYE (bar group 4), only a negligible fraction of sites had no horseshoe-shaped Bassoon under both conditions. The total number of horseshoe-shaped Bassoon sites in EGTA-treated retinae was reduced in the same OPL volume. **F**, **G:** Representative images of the OPL double labeled for the rod photoreceptor terminal marker Veli3 (magenta) and Bassoon (green) from untreated (**F**) and EGTA-treated (**G**) retinae. In the EGTA-treated retina, Veli3 labeled rod photoreceptor terminals often show punctate or a lack of Bassoon labeling, which is in contrast to the horseshoe-shaped Bassoon labeling present in rod photoreceptor terminals of untreated retina. Scale bars: 5 µm (**A**, **B**, **F**, **G**), 0.2 µm (**C**, **D**).

We next processed untreated, ionophore-treated, and EGTA-treated retinae for light microscopy and compared ribbon morphology in rod photoreceptor terminals using immunocytochemistry. In the first step, we compared the localization of ribbon material, stained for the main ribbon constituent RIBEYE [24,25], with staining for Bassoon, a protein anchoring the ribbon to the arciform density/plasma membrane compartment (Figure 2) [26,27].

Under untreated and ionophore-treated conditions, most rod photoreceptor ribbons showed a horseshoe-shaped staining for RIBEYE, which bent around the Bassoon staining (Figure 2A). In contrast, EGTA treatment affected the localization of RIBEYE at the active zone (Figure 2B). In many rod photoreceptor terminals, RIBEYE disintegrated into several aggregates, which were associated with the regular horseshoe-shaped Bassoon (Figure 2B), a finding corroborated by pre-embedding immunoelectron microscopy (Figure 2C, arrowhead). Bassoon, on the other hand, stayed at its regular localization along the arciform density (Figure 2D, arrows). It must be noted that under the same conditions, we also found rod photoreceptor ribbon synaptic complexes with a more progressed state of disassembly. At such synaptic sites, RIBEYE appeared disintegrated; also, the Bassoon staining was less delineated. The observed morphological heterogeneity most likely represents snapshots of a dynamic process of ribbon synapse disassembly.

For a more detailed analysis, we quantified the various morphological ribbon states (Figure 2E). We first defined intact rod photoreceptor active zones as horseshoe-shaped structures (HS), either stained by Bassoon (Figure 2E, bar groups 1–3) or RIBEYE (Figure 2E, bar group 4). Under untreated conditions, 95% of rod photoreceptor active zones, defined by a horseshoe-shaped Bassoon staining, were associated with horseshoe-shaped RIBEYE staining. In EGTA-treated retinae, this fraction was reduced and only 61% of the horseshoe-shaped Bassoon staining was associated with regular horseshoe-shaped RIBEYE staining. The remaining horseshoe-shaped Bassoon-stained active zones were associated with RIBEYE aggregates (club-shaped, CS, and spherical-shaped, SS) or diffuse RIBEYE (Figure 2E). On the other hand, when we examined active zones defined by horseshoe-shaped RIBEYE staining, we found only a negligible fraction that had no horseshoe-shaped Bassoon staining, both under untreated and EGTA-treated conditions (0.3% and 0.5%, respectively). Interestingly, the total number of detectable horseshoe-shaped Bassoon-stained active zones, counted from the same volume of OPL, was markedly reduced in EGTA-treated retinae when compared with control retinae (671 versus 1,293). The reduction in active zones following EGTA treatment was not caused by a general loss of photoreceptor terminals, as seen in experiments in which photoreceptor terminals were labeled with the marker Veli3 [28,29], but by the selective loss of active zones. We often found rod photoreceptor terminals outlined by Veli3 immunoreactivity with only diffuse or dot-like Bassoon labeling (Figure 2G), whereas under control conditions, horseshoe-shaped Bassoon labeling always lay within the Veli3-stained terminals (Figure 2F).

In summary, the results suggest that Bassoon is a more stable component of the presynaptic ribbon complex than RIBEYE. Ribbon complex disassembly seems to be a sequential process, beginning with the initial removal of ribbon material and the subsequent disintegration of Bassoon localization.

### Redistribution of ribbon and arciform density/plasma membrane proteins

**Figure 3:**
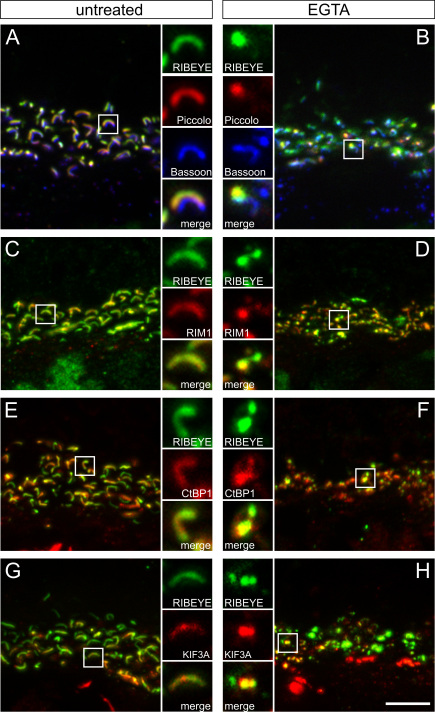
Synaptic ribbon proteins co-distribute with RIBEYE. **A**, **B**: Representative images of the outer plexiform layer (OPL) with the photoreceptor ribbon synapses triple labeled for RIBEYE (green), Piccolo (red), and Bassoon (blue) from untreated (**A**) and EGTA-treated (**B**) retinae. Under EGTA treatment, both RIBEYE and Piccolo change from a horseshoe-shaped to a punctate appearance, even when a Bassoon horseshoe shape is still visible (**B**). **C-H**: Representative images of the OPL with the photoreceptor ribbon synapses double labeled for RIBEYE (green) and ribbon associated proteins (red) from untreated (**C**, **E**, **G**) and EGTA-treated (**D**, **F**, **H**) retinae. Like RIBEYE and Piccolo, RIM1, CtBP1, and KIF3A also change from a horseshoe-shaped to a punctate appearance under EGTA treatment and co-localize with the RIBEYE aggregates. Scale bar: 5 µm.

**Figure 4:**
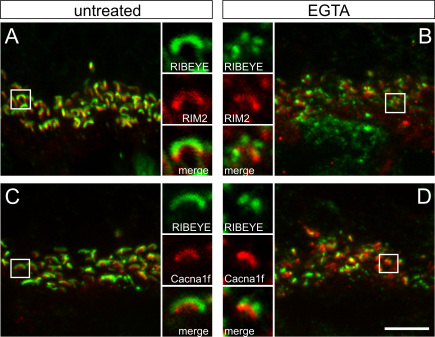
Arciform density/plasma membrane proteins are stable components at the ribbon synaptic site. **A-D**: Representative images of the outer plexiform layer with the photoreceptor ribbon synapses double labeled for RIBEYE (green) and components of the arciform density/plasma membrane compartment (red) from untreated (**A**, **C**) and EGTA-treated (**B**, **D**) retinae. The arciform density/plasma membrane proteins RIM2 (**B**) and the L-type Ca^2+^ channel subunit Cacna1f (**D**) often show a horseshoe-shaped labeling under EGTA treatment, when RIBEYE has already disintegrated into aggregates. Scale bar: 5 µm.

In a previous study, we assigned several proteins with horseshoe-shaped labeling at the rod photoreceptor ribbon synapse to two sub-compartments within the ribbon complex [27]. The molecular components of these sub-compartments were separated into ribbon-associated proteins (Piccolo, RIM1, CtBP1, and KIF3A) and arciform density/plasma membrane proteins (RIM2, Munc13, CAST1, and the L-type Ca^2+^ channel subunit Cacna1f), based on their co-localization with RIBEYE in the retina of a mutant mouse with a defect in ribbon anchorage [26,27]. In the next step, we therefore examined whether the characteristic morphological changes evoked by EGTA treatment are specific to RIBEYE and Bassoon or instead involve the whole set of proteins of the two sub-compartments (Figure 3 and Figure 4).

Labeling against the ribbon-associated proteins Piccolo, RIM1, CtBP1, and KIF3A revealed a horseshoe-shaped staining pattern in rod photoreceptor ribbon synapses under untreated conditions (Figure 3A,C,E,G), as well as under ionophore conditions (not shown). These proteins were redistributed at many synapses after EGTA treatment (Figure 3B,D,F,H). Under EGTA treatment, Piccolo, RIM1, CtBP1, and KIF3A clearly co-localized with the RIBEYE aggregates (Figure 3B,D,F,H), even when Bassoon still showed a horseshoe-shaped staining pattern (Figure 3B).

Unlike the ribbon-associated proteins, the arciform density/plasma membrane proteins RIM2 and the L-type Ca^2+^ channel subunit Cacna1f did not show prominent differences in their staining pattern in the untreated (Figure 4A,C), ionophore-treated (not shown), or EGTA-treated (Figure 4B,D) retinae. Double labeling with RIBEYE revealed that in many ribbon synapses of EGTA-treated retinae, stable horseshoe-shaped staining of RIM2 (Figure 4B) and the L-type Ca^2+^ channel subunit Cacna1f (Figure 4D) was present, although in the same synapses, RIBEYE had already disintegrated into several aggregates. Only occasionally, we observed rod photoreceptor terminals in which both RIM2 and the L-type Ca^2+^ channel subunit Cacna1f were diffusely distributed or almost undetectable (not shown).

## Discussion

In vivo studies using albinotic BALB/c mice showed that rod photoreceptor ribbons undergo illumination-dependent structural changes: ribbon material is lost during light exposure and regained in the dark [1,5]. As the molecular basis of these changes is still unclear, the aim of this study was to relate the structural ribbon changes to the redistribution of known proteins of the presynaptic ribbon complex. To experimentally disassemble the photoreceptor presynaptic ribbon complex, we adapted the in vitro assay described by Spiwoks-Becker and colleagues [5] using an ionophore and EGTA to manipulate the Ca^2+^ concentrations.

### Presynaptic ribbon complex disassembly occurs in two steps

**Figure 5:**
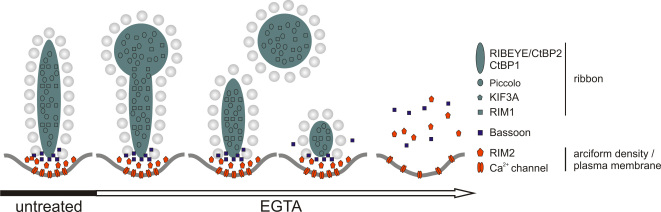
Scheme of the redistribution of proteins of the presynaptic ribbon complex during EGTA treatment.

The staining patterns of molecular components of the presynaptic ribbon complex in control and EGTA-treated retinae support the assumption that spherical material pinching off the ribbon complex is initially bona fide ribbon material, including RIBEYE, Piccolo, CtBP1, RIM1, and KIF3A. The light microscopic appearance of spherical profiles correlated well with the spherical electron dense material observed with electron microscopy. However, when ribbon material pinches off, the arciform density and plasma membrane components Bassoon, RIM2, and the L-type Ca^2+^ channel subunit Cacna1f still mark the transmitter release sites. This compartment seems to be a rather stable component of the ribbon synaptic complex (Figure 5), and could serve as a template/scaffold to rebuild the ribbon complex during adaptation dependent remodeling of the ribbon, viz. elongation of the ribbon in darkness. Nonetheless, the extent of the effects elicited by EGTA treatment varied between individual retinae and photoreceptor terminals, and the remodeling process did not necessarily stop at the level of ribbon sphere formation. Sometimes, ribbon proteins adopted a cloudy distribution within the terminal. Only at this stage, arciform density/plasma membrane protein localization started to disintegrate (Figure 5).

### Synaptic spheres: common modules in ribbon turnover, degradation, and development

The course of ribbon disassembly following EGTA treatment described in this study seems to happen under physiologic as well as pathological conditions. Disassembly begins with the formation of protrusions and the pinching off of spherical ribbon material.

### Ribbon turnover

Extensive formation of protrusions and spheres was observed in studies in vivo and in vitro using albinotic BALB/c mice [1,5]. In vivo, light triggered the process and darkness reversed it. Light exposure activates the phototransduction mechanism, which finally leads to a decrease in the intracellular Ca^2+^ concentration and the hyperpolarization of the photoreceptor membrane. As vesicle exocytosis from photoreceptor ribbon synapses involves voltage-dependent L-type Ca^2+^ channels, the synaptic Ca^2+^ concentration is also decreased and transmitter release is reduced. In vitro, the addition of the Ca^2+^ chelators EGTA or BAPTA to the incubation medium, in which the explanted, dark-adapted retinae were kept, led to significantly smaller ribbons and to the occurrence of a large number of synaptic spheres [5]. Furthermore, increasing the intracellular Ca^2+^ concentration by applying a Ca^2+^ ionophore and CaCl_2_ before light exposure prevented the light effects on the ribbon structure, viz. the formation of protrusions and synaptic spheres [5]. The results from the studies in the retina of albino [1,5] and pigmented mice (this study) suggest that the structural ribbon changes are caused by a mechanism that depends on synaptic activity and the intracellular Ca^2+^ concentration. Ca^2+^ has also been shown to regulate ribbon synaptic morphology in cone photoreceptor cells of teleost fish [18]. How Ca^2+^ and synaptic activity cause the structural changes is unknown, as we do not know where Ca^2+^ exerts its effects.

### Ribbon degradation

Changes in ribbon morphology and particularly the occurrence of synaptic spheres are signs of pathophysiological processes caused by malfunctioning ribbon synapses. For example, in photoreceptor terminals of a mouse retina deficient of the presynaptic cytomatrix protein Bassoon, a major component of the ribbon complex, ribbons are not anchored to the presynaptic active zone, and rapidly disintegrate into synaptic spheres [26,30]. The functionally impaired cone photoreceptor ribbon synapses of the zebrafish *no optokinetic response c* mutant display a similar ribbon synaptic phenotype with free-floating ribbons and spherical ribbon densities [19]. Recently, we reported the occurrence of synaptic spheres in photoreceptor terminals of the Cplx3/4 double-knockout retina [31]. Complexins are SNARE regulatory proteins, and the lack of Cplx3 and Cplx4, which are specifically expressed at retinal ribbon synapses, perturbs ribbon synaptic function [31,32]. The synaptic spheres seen in the Cplx3/4 double-knockout retina are most likely ribbon breakdown products as a consequence of the altered synaptic activity.

### Ribbon development

Finally, synaptic spheres have been described during photoreceptor ribbon development. We recently reported that spheres are the transport units for proteins of the cytomatrix at the active zone in the assembly of the photoreceptor ribbons during synaptogenesis [33–35]. These non-membranous, electron-dense precursor spheres contain proteins like Bassoon, Piccolo, RIBEYE, and RIM1, but not proteins of the arciform density/plasma membrane like Munc13, CAST1, RIM2, and the L-type Ca^2+^ channel subunit Cacna1f.

In conclusion, synaptic spheres are common modules in ribbon dynamics and are involved in such different processes as turnover, degradation, and development. Of the known proteins, RIBEYE is most likely a main contributor in the process of ribbon assembly and disassembly. In accordance with this finding, multiple RIBEYE-RIBEYE interactions were recently described and suggested to create a dynamic scaffold of the synaptic ribbon [36]. Whether the described processes utilize similar or different molecular mechanisms is hard to predict, as we still know too little about the function of the many proteins constituting the presynaptic ribbon complex.
